# Effects of exercise intensity and frequency on improving cognitive performance in middle-aged and older adults with mild cognitive impairment: A pilot randomized controlled trial on the minimum physical activity recommendation from WHO

**DOI:** 10.3389/fphys.2022.1021428

**Published:** 2022-09-19

**Authors:** Danny J. Yu, Angus P. Yu, Joshua D. K. Bernal, Daniel Y. Fong, Derwin K. C. Chan, Calvin P. Cheng, Parco M. Siu

**Affiliations:** ^1^ Division of Kinesiology, School of Public Health, Li Ka Shing Faculty of Medicine, The University of Hong Kong, Pokfulam, Hong Kong SAR, China; ^2^ School of Nursing, Li Ka Shing Faculty of Medicine, The University of Hong Kong, Pokfulam, Hong Kong SAR, China; ^3^ Department of Early Childhood Education, Faculty of Education and Human Development, The Education University of Hong Kong, Pokfulam, Hong Kong SAR, China; ^4^ Department of Psychiatry, Li Ka Shing Faculty of Medicine, The University of Hong Kong, Pokfulam, Hong Kong SAR, China; ^5^ Department of Psychiatry, Queen Mary Hospital, Pokfulam, Hong Kong SAR, China

**Keywords:** mild cognitive impairment, cognitive performance, walking exercise, exercise intensity, exercise frequency

## Abstract

**Background:** The World Health Organization physical activity guidelines recommend adults and older adults to accumulate at least 150–300 min of moderate or 75–150 min of vigorous aerobic-type physical activity weekly for health benefits including improvements of cognitive performance. However, the optimal exercise intensity and frequency for maximizing the cognitive benefits remain unclear.

**Purpose:** We conducted a parallel, assessor-blinded, pilot randomized controlled trial to evaluate the effectiveness of different intensities and frequencies of the WHO-recommended minimal volume of aerobic-type physical activity on improving cognitive performance in middle-aged and older adults with mild cognitive impairment (MCI).

**Methods:** Participants were randomly allocated to the stretching exercise control group (CON), once-a-week and thrice-a-week moderate-intensity walking groups (M1 and M3), and once-a-week and thrice-a-week vigorous-intensity walking groups (V1 and V3). Intervention duration was 12 weeks. The primary outcome was global cognitive performance assessed by the Hong Kong version of Montreal Cognitive Assessment. Secondary outcomes were self-report and objective cognitive performances, mental health, sleep quality, and cardiorespiratory fitness.

**Results:** Thirty-seven participants completed the study (CON: *n* = 7, M1: *n* = 7, M3: *n* = 7, V1: *n* = 8, V3: *n* = 8). Participants in all four walking exercise groups demonstrated significant improvements in global cognitive performance assessed by the Hong Kong version of the Montreal Cognitive Assessment after the intervention when compared to CON (*p* < 0.001). The walking exercise interventions also significantly mitigated the anxiety severity (*p* < 0.005) and improved the cardiorespiratory fitness (*p* < 0.05) of the participants in the walking exercise groups.

**Conclusion:** 150-min moderate- or 75-min vigorous-intensity walking exercise performed once- or thrice-weekly showed similar effects on improving cognitive performance in middle-aged and older adults with MCI. The 12-week walking exercise interventions also reduced anxiety severity and improved cardiorespiratory fitness of the participants.

**Clinical Trial Registration:** clinicaltrials.gov, identifier NCT04515563

## 1 Introduction

Dementia is one of the major causes of disability and mortality worldwide (Shah et al., 2016). The World Health Organization (WHO) reported that over 55 million people were living with dementia in 2021, and this number is expected to triple by 2050 (World Health Organization 2017). Mild cognitive impairment (MCI) is recognized as a transitional phase in the early stage of dementia, where patients present with memory loss or declines in other cognitive domains, such as attention and processing speed, while the daily independent living of the patients remain intact (Petersen and Knopman 2006). Regarded as the prodromal stage of dementia, MCI is a critical timeframe to intervene to decelerate or even reverse the cognitive deterioration, and to prevent the development of dementia (Petersen et al., 2018). The prevalence of MCI is expected to increase with an aging global population. Indeed, MCI is common among older adults, with a prevalence ranging from 10% to 20% (Langa and Levine 2014). Alarmingly, the annual conversion rate of MCI to dementia ranges from 5% to 10% (Mitchell and Shiri-Feshki 2009), which highlights the urgent need to identify effective interventions to slow down or prevent the progression of MCI to dementia.

Although currently there is no effective pharmacological treatment for MCI(Petersen et al., 2018), exercise has been demonstrated to be an effective non-pharmacological approach to alleviate cognitive deterioration in MCI patients (Zheng et al., 2016; Northey et al., 2018). In 2018, the American Academy of Neurology (AAN) updated the practice guidelines on the diagnosis and treatment of MCI, which recommends regular exercise (twice a week) as part of the overall management approach (Petersen et al., 2018). Nevertheless, it is worth noting that the recommendation on the frequency of exercise was based on two randomized controlled trials that could be classed as moderate-level evidence (Nagamatsu et al., 2012; Suzuki et al., 2013). Whether twice weekly is the minimal or optimal exercise frequency for maximizing cognitive benefits in patients with MCI remains unknown. Moreover, the AAN guidelines on MCI do not provide any recommendations on exercise intensity, likely due to the lack of rigorous investigations evaluating the effectiveness of exercise intensity on treating MCI. A review conducted by Northey and colleagues advocated that future studies should move beyond investigating the effectiveness of exercise interventions to optimizing the exercise prescription parameters such as exercise frequency and intensity to maximize the cognitive benefits (Northey et al., 2018).

The WHO physical activity guidelines recommend adults and older adults should perform a minimum of 150–300 min of moderate-intensity or 75–150 min of vigorous-intensity physical activity weekly (or an equivalent combination of both) to gain health benefits such as improvements of cognitive health (Bull et al., 2020). However, the impact of exercise frequency and intensity in exercise interventions adopting the WHO recommendations for improving cognitive performance in MCI patients has not been investigated in a randomized controlled trial setting.

As one of the most commonly practiced exercise modalities, walking exercise is free, safe, and confers various health benefits, including improvement of cognitive performance (Sitthipornvorakul et al., 2018; Doyle et al., 2019; Tang et al., 2019; Barha et al., 2022). More importantly, walking exercise is well recognized as a suitable exercise modality for the physically inactive unfit individuals and older adults (Cohen-Mansfield et al., 2004). Utilizing walking exercise as the intervention modality, the present study aimed to 1) investigate the effectiveness of a 12-week walking exercise intervention following the WHO physical activity guidelines on improving cognitive performance in middle-aged and older adults with MCI, 2) compare the effectiveness of moderate- and vigorous-intensity walking exercise on improving cognitive performance in middle-aged and older adults with MCI, and 3) compare the effectiveness of once-a-week and thrice-a-week walking exercise on improving cognitive performance in middle-aged and older adults with MCI.

## 2 Materials and methods

### 2.1 Participants

This was a single-center, five-arm, parallel, assessor-blinded, pilot randomized controlled trial conducted in The University of Hong Kong between August 2020 and September 2021. Participants were recruited through advertisements in community centers, housing estates, and local universities. A total of 312 individuals were interested in participating in the study and were invited for eligibility screening. The inclusion criteria included: 1) aged ≥50 years, 2) ethnic Chinese, 3) diagnosed with MCI (amnestic and non-amnestic MCI) according to the Mayo Clinic diagnostic criteria (i.e., patient-reported subjective decline of cognitive function and the total score of the Hong Kong version of Montreal Cognitive Assessment was between the 2nd and the 7th percentile for age- and education-corrected normative data in Hong Kong (Knopman and Petersen 2014; Yeung et al., 2014; Wong et al., 2015); and 4) normal daily functioning score ≥2 points (on a 4-point scale) in every item of the Chinese Lawton Instrumental Activities of Daily Living Scale (Tong and Man 2002). The exclusion criteria included: 1) living with serious somatic conditions (such as loss of limbs) that prevent participation in walking exercises, 2) unable to walk without an assistive device, 3) presenting with or had a history of major diseases such as cancer, 4) diagnosed with neurodegenerative diseases such as Alzheimer’s disease, and 5) having regular exercise habit (defined as exercise for ≥50 min more than three times per week). Overall, 50 eligible participants enrolled in the study after giving written informed consent. Participants were provided with both written and verbal information about the study, including the benefits and risks. This study was approved by The University of Hong Kong/Hospital Authority Hong Kong West Institutional Review Board (approval number: UW 20-366).

### 2.2 Randomization and masking

The randomization sequence was generated with a block size of five using an online randomization website (https://www.sealedenvelope.com/randomisation/internet/). The randomization was performed by an independent researcher who was not involved in participant recruitment. The randomization sequence was concealed from the researcher responsible for participant allocation. Participants were randomly allocated to the stretching exercise control group (CON), once-a-week moderate-intensity walking group (M1), thrice-a-week moderate-intensity walking group (M3), once-a-week vigorous-intensity walking group (V1), and thrice-a-week vigorous-intensity walking group (V3) on 1:1:1:1:1 ratio. The outcome assessors were blinded to the participants’ group allocation. Due to the nature of the interventions, exercise instructors were not blinded to the group allocations.

### 2.3 Intervention

The walking exercise training was implemented under the supervision of research personnel for 12 weeks in the research center. The intervention was conducted individually and there was no social interaction between the participants.

#### 2.3.1 Exercise intensity

The WHO physical activity guidelines recommend (on an absolute scale) moderate-intensity exercise to be performed at 3.0–5.9 times the intensity of resting [3.0–5.9 metabolic equivalents of task (METs)] and vigorous intensity at 6.0 METs or above. The exercise intensity was set at 3.5 METs for moderate-intensity walking groups and 7 METs for vigorous-intensity walking groups in this study.

#### 2.3.2 Exercise frequency

Exercise frequency was set at once or three times weekly.

#### 2.3.3 Exercise volume

Adopting the minimal exercise volume suggested by the WHO physical activity guidelines, the overall duration of the exercise sessions was set at 150 min for moderate-intensity walking groups (150 min each session in M1 and 50 min each session in M3) and 75 min for vigorous-intensity walking groups (75 min each session in V1 and 25 min each session in V3).

#### 2.3.4 Training implementation

Before each session, there was a 5-min warm-up period, in which participants performed static stretching exercises of the major muscle groups including chest, back, and lower limbs. Participants were then instructed to walk on a treadmill with the speed and inclination gradually increased to achieve the target heart rate (THR) range within 5 min. The THR was calculated according to the linear relationship between heart rate and oxygen consumption (VO_2_) recorded in the maximal oxygen consumption (VO_2max_) treadmill test (Achten and Jeukendrup 2003). The data of heart rate and VO_2_ were exported into Excel, with VO_2_ as the independent variable, and heart rate as the dependent variable. The linear equation between the VO_2_ and the heart rate was computed using the “Display Equation on chart” function. Using the linear equation and the exercise intensity (3.5 METs or 7 METs) assigned to the specific participant, the THR was then calculated (Yu et al., 2022b). Participants were required to maintain the heart rate within the THR range (THR ±10 beats per minute) during the walking sessions. A validated heart rate monitor (Polar OH1) was used to monitor heart rate (Hettiarachchi et al., 2019). At the end of each session, there was a 5-min cool-down period, in which the speed and inclination of the treadmill was gradually decreased, followed by static stretching exercises. As the duration of session in M1 and V1 were longer, participants were allowed two 10-min breaks to rehydrate and rest during the training sessions. The weekly exercise volumes of the four walking exercise groups were equivalent (525 MET-Mins).

#### 2.3.5 Intervention for the control group

During the 12-week intervention period, participants in the control group (CON) received once-a-week individual stretching exercise intervention, which covered the major muscle groups such as chest, back, and lower limbs. The session duration of the CON intervention was 75 min matching to weekly exercise duration of V1.

All participants were instructed to maintain their lifestyle routines throughout the study period, including their normal habitual physical activity.

### 2.4 Sample size estimation

Estimation of sample size was performed using G*Power 3.0 by setting the test family and statistical test to “F tests” and “ANOVA: Repeated measures, within-between interaction”. Based on a medium effect size of interaction Cohen’s d = 0.3 and a five-arm pretest-posttest design, 40 participants were needed to achieve an 80% statistical power with *α* = 0.05 (Heyn et al., 2004; Yu et al., 2022b). Considering a 15% drop-out rate, a total of 50 participants (rounded up from 48) were required (Yu et al., 2022a).

### 2.5 Outcome measures

#### 2.5.1 Primary outcome

The primary outcome was global cognitive performance measured by the Hong Kong version of Montreal Cognitive Assessment (HK-MoCA). The HK-MoCA is a validated cognitive assessment that takes into account different domains of cognitive performance, which can examine the cognitive profile of the individuals (Yeung et al., 2014). The inter-rater reliability of the HK-MoCA was 0.99, and the Cronbach’s alpha score was 0.77, indicating an acceptable internal consistency (Wong et al., 2009). The HK-MoCA was also demonstrated to have a high level of validity to differentiate MCI and dementia patients from individuals with normal cognition, with a sensitivity of 0.93, and specificity of 0.74 (Wong et al., 2009). A higher score of HK-MoCA indicates better global cognitive performance. An improvement ≥4 points in HK-MoCA is defined as the minimal clinically important difference (MCID) (Feeney et al., 2016; Kopecek et al., 2017).

#### 2.5.2 Secondary outcomes

Self-report cognitive performance was assessed by the Cognitive Self-Report Questionnaire (CSRQ) (Leung et al., 2018). The CSRQ has been validated in Hong Kong and comprises 20 questions with an overall score ranging from 0-100. A higher score indicates worse self-perception on cognitive performance (Leung et al., 2018). The CSRQ was demonstrated to have good internal consistency (*α* = 0.91) and 2-month test–retest reliability (r = 0.85) (Spina et al., 2006; Leung et al., 2018).

Objective cognitive performance was measured by the computerized National Institutes of Health (NIH) Toolbox - Cognition Battery. Five tests including Flanker inhibitory control and attention test, dimensional change card sort test, picture sequence memory test, pattern comparison processing speed test, and oral symbol digit test were selected from the Toolbox to assess the performances of different cognitive domains, including attention, inhibitory control, cognitive flexibility, executive function, episodic memory and processing speed. After finishing the tests, a total composite score of the five tests was generated by the Toolbox, which reflected the overall objective cognitive performance. A higher composite score indicates better objective cognitive performance (Akshoomoff et al., 2013; Weintraub et al., 2013; Heaton et al., 2014). The NIH Toolbox—Cognition Battery showed excellent test-retest reliability (r: 0.86–0.92) and internal consistency (Cronbach’s alpha score 0.77–0.84) (Heaton et al., 2014).

Cardiorespiratory fitness (VO_2max_) was measured by the symptom-free cardiorespiratory treadmill test using a modified Bruce protocol (Yu et al., 2022b). The VO_2max_ responses were determined as meeting at least two of the following criteria: 1) plateau of VO_2_ with increasing intensity, 2) respiratory exchange ratio (RER) ≥1.10, and 3) heart rate ≥95% age-predicted maximal heart rate (220-age) (Yu et al., 2022b).

2.5.2.4 Mental health including severity of depression and anxiety was assessed by the Hospital Anxiety and Depression Scale (HADS) (Leung et al., 1999). This questionnaire has two 7-item subscales for both depression and anxiety and the overall score ranges from 0-21, with a higher score indicating more severe depressive/anxious symptoms. The Chinese version has been demonstrated to be reliable and valid in Hong Kong Chinese (Leung et al., 1999).

Subjective sleep quality and quantity were assessed by the Pittsburgh Sleep Quality Index (PSQI) (Tsai et al., 2005). This instrument contains 19 items that subjectively assess sleep quality, sleep quantity, perceived restfulness, and sleep disturbances by measuring usual bedtime, wake time, time to fall asleep, and time of actual sleep, etc. A higher score indicates poorer sleep quality (Tsai et al., 2005).

Body weight and height were measured by validated weighing scales (A&D UC-321) and stadiometer (SECA 213).

All the outcome measurements were conducted at baseline and after the 12-week intervention.

### 2.6 Data analysis

Data were presented as mean and standard deviation. Data were analyzed by generalized estimating equation (GEE) using group and time as main effects and baseline as a covariate (Siu et al., 2021; Yu et al., 2022a; Chin et al., 2022). A significant group-by-time interaction indicated that there was a significant difference in the intervention-mediated changes among groups by time. When a significant group-by-time interaction was observed, pairwise comparison was performed to compare the differences between the intervention groups using a closed test procedure followed by Bonferroni-Holm correction to correct for multiple comparisons (Siu et al., 2021). Statistical significance was considered at *p* < 0.05. All statistical analyses were performed using R, version 4.1.0.

## 3 Results

### 3.1 Baseline characteristics of participants

**FIGURE 1:**
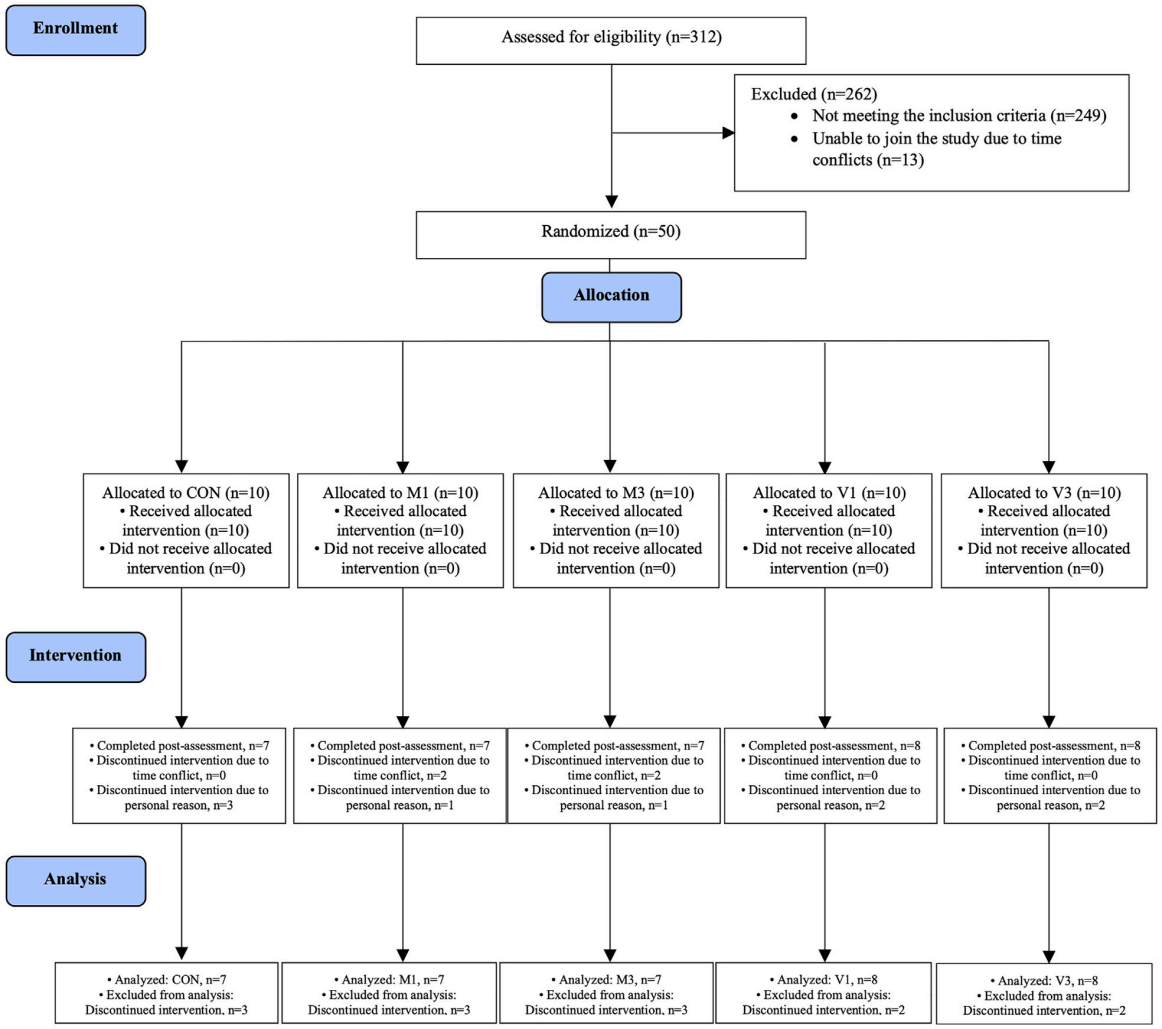
Schematic representation of participants screening, randomization and interventions.

A total of 312 individuals were screened between August 2020 and September 2021. Fifty eligible participants were randomly allocated to CON (*n* = 10), M1 (*n* = 10), M3 (*n* = 10), V1 (*n* = 10), and V3 (*n* = 10) groups (Figure 1). All intervention sessions were started within 2 weeks after the baseline assessments to ensure baseline data validity. The post-intervention assessments were performed within 2 weeks after the last training session to ensure data validity. A total of 37 participants completed the 12-week intervention and the post-intervention assessments (CON: *n* = 7, M1: *n* = 7, M3: *n* = 7, V1: *n* = 8, V3: *n* = 8). The intervention adherence rates as determined by session attendance rates were 91.7%, 94.4%, 95.2%, 94.8%, and 97.6% in CON, M1, M3, V1, and V3, respectively. The baseline characteristics are summarized in Table 1.

**TABLE 1:** Baseline characteristics of participants.

Group	CON	M1	M3	V1	V3
Number of Participants	7	7	7	8	8
Gender, F:M	6:1	6:1	6:1	7:1	8:0
Age, year	63.7 (4.7)	63.5 (7.0)	63.5 (5.7)	63.4 (5.2)	63.3 (5.1)
Weight, kg	55.8 (11)	57.4 (8.9)	65.7 (7.1)	57.3 (8.2)	59.8 (11.1)
Height, cm	154.8 (7.3)	156.7 (8.9)	157.1 (7.4)	158.5 (7.2)	158.7 (5.9)
High school education or above, n	5	5	5	6	6
Marital status, n					
Single	0	1	1	0	2
Married	7	5	6	7	5
Divorced	0	1	0	1	0
Widowed	0	0	0	0	1
Current cigarette smoking, n	0	0	0	0	1
Current alcohol drinking, n	0	0	0	0	0
Hong Kong- montreal cognitive assessment, total score	19.9 (3.5)	19.7 (1.9)	18.9 (1.9)	20.1 (1.7)	19.6 (2.5)
Cognitive self-report questionnaire, total score	73 (11.3)	74.6 (7.5)	79.4 (14.3)	69.3 (18.0)	73.5 (15.3)
NIH toolbox - cognition battery, total score	435.1 (85)	440.8 (73.4)	445.4 (38.7)	470.7 (38.7)	451.7 (30.2)
VO_2max_, mL/kg/min	32.4 (4.1)	26.9 (5.1)	28.2 (3.8)	32 (6.0)	29.6 (4.5)
Hospital anxiety and depression scale					
Anxiety subscale, score	6.0 (3.5)	5.1 (2.8)	7.9 (4.5)	5.3 (4.1)	5.3 (1.7)
Depression subscale, score	6.9 (3.5)	6.3 (2.2)	10.9 (1.9)	6.3 (3.4)	7.5 (4.7)
Pittsburgh sleep quality index, total score	11.0 (3.5)	10.9 (2.1)	9.7 (4.3)	9.9 (3.5)	13.1 (3.5)
Average target heart rate, beats/min	N/A	106.7 (14.6)	100.7 (12.9)	129.0 (16.2)	131.1 (6.9)

Data are expressed as mean (standard deviation). CON, stretching exercise control group, M1, moderate-intensity once-a-week walking group, M3, moderate-intensity thrice-a-week walking group, V1, vigorous-intensity once-a-week walking group, and V3, vigorous-intensity thrice-a-week walking group.

### 3.2 Primary outcome

**FIGURE 2:**
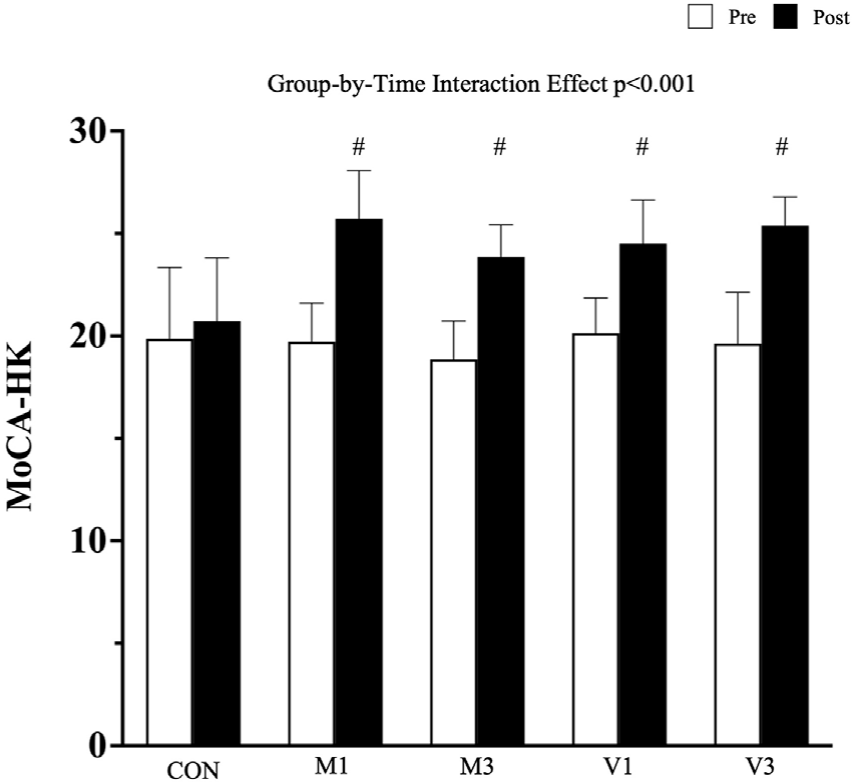
Global cognitive Performance Measured by HK-MoCA. Data are expressed as mean (standard deviation). CON: stretching exercise control group, M1: moderate-intensity once-a-week walking group, M3: moderate-intensity thrice-a-week walking group, V1: vigorous-intensity once-a-week walking group, and V3: vigorous-intensity thrice-a-week walking group. #: *p* < 0.001 compared with CON at the post-intervention measurement.

A significant group-by-time interaction effect was observed in the global cognitive performance measured by HK-MoCA (*p* < 0.001). After the 12-week walking intervention, HK-MoCA scores significantly increased in M1, M3, V1, and V3 compared with CON [adjusted mean difference (95% CI): M1 vs. CON: 5.1 (3.5, 6.7), *p* < 0.001; M3 vs. CON: 3.9 (2.3, 5.5), *p* < 0.001; V1 vs. CON: 3.6 (2.1, 5.1), *p* < 0.001; and V3 vs. CON: 4.8 (3.3, 6.4), *p* < 0.001]. No significant difference was found among the four walking exercise groups in the post hoc analysis (all *p* > 0.05) (Figure 2).

### 3.3 Secondary outcomes

**FIGURE 3:**
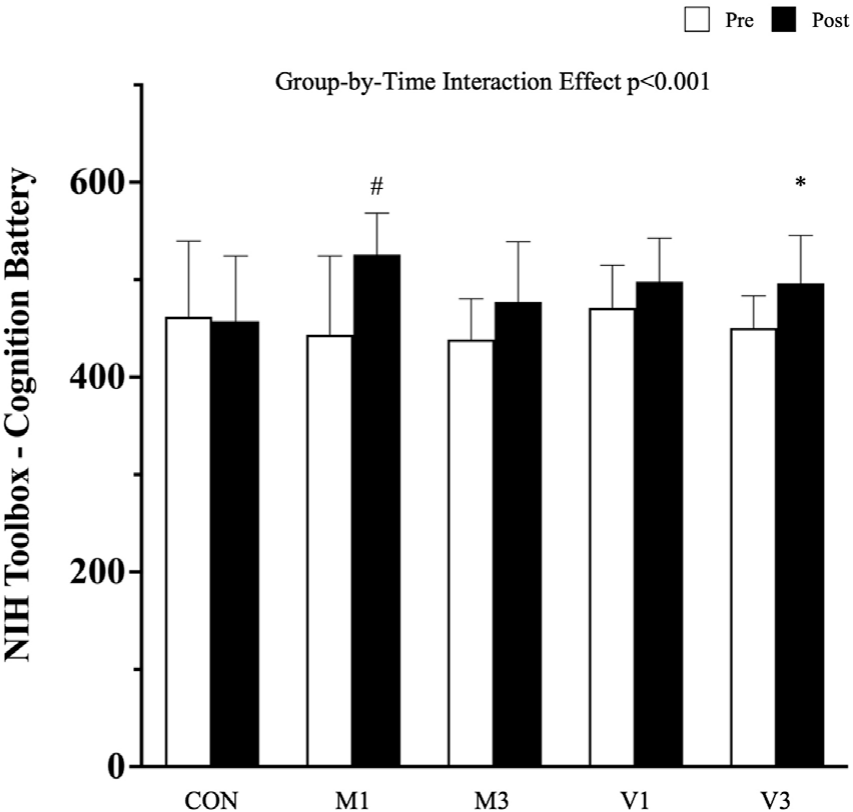
Cognitive performance measured by NIH toolbox-cognition battery. Data are expressed as mean (standard deviation). CON: stretching exercise control group, M1: moderate-intensity once-a-week walking group, M3: moderate-intensity thrice-a-week walking group, V1: vigorous-intensity once-a-week walking group, and V3: vigorous-intensity thrice-a-week walking group. #: *p* < 0.001 compared with CON at the post-intervention measurement; *: *p* < 0.05 compared with CON at the post-intervention measurement.

A significant group-by-time interaction effect was observed in the objective cognitive performance measured by the NIH Toolbox - Cognition Battery (*p* < 0.01). After the 12-week walking intervention, the total composite scores in NIH Toolbox - Cognition Battery were significantly increased in M1 and V3 compared with CON [adjusted mean difference (95% CI): M1 vs. CON: 83.5 (51.1, 115.9), *p* < 0.001; and V3 vs. CON: 48.3 (16.9, 79.6), *p* = 0.013], whereas an improving trend was detected in M3 and V1 [M3 vs. CON: 38.6 (6.2, 71.10, *p* = 0.056; V1 vs. CON: 33.5 (2.2, 64.8), *p* = 0.081], although this was not statistically significant (Figure 3).

A significant group-by-time interaction effect was observed in the VO_2max_ test (*p* < 0.05). After the 12-week walking intervention, VO_2max_ was significantly increased in M1, M3, V1, and V3 compared with CON [adjusted mean difference (95% CI): M1 vs. CON: 4.3 (2.9, 5.8), *p* < 0.001; M3 vs. CON: 3.4 (1.9, 4.8), *p* < 0.001; V1 vs. CON: 3.7 (2.3, 5.0), *p* < 0.001; and V3 vs. CON: 4.5 (3.1, 5.9), *p* < 0.001] (Table 2).

**TABLE 2:** Summary of secondary outcomes.

	Baseline (0 week)	Post-intervention (12 weeks)	Interaction effect	Group effect	Time effect		ΔMean_adj_ [95%CI]	*p* value
Cognitive self-report questionnaire								
CON	73.0 (11.3)	75.4 (12.3)	0.119	0.215	0.713		N/A	N/A
M1	74.6 (7.5)	67.0 (5.8)						
M3	79.4 (14.3)	73.0 (13.4)						
V1	69.3 (18)	60.3 (12.5)						
V3	73.5 (15.3)	64.9 (13.4)						
Cardiopulmonary VO_2max_ test								
CON	32.4 (4.1)	31.2 (3.2)	<0.05	0.146	0.767			
M1	26.9 (5.1)	30.3 (5.3)				M1 vs. CON	4.3 (2.9 to 5.8)	<0.001
M3	28.2 (3.8)	30.6 (4.4)				M3 vs. CON	3.4 (1.9 to 4.8)	<0.001
V1	32.0 (6.0)	34.4 (5.6)				V1 vs. CON	3.7 (2.3 to 5.0)	<0.001
V3	29.6 (4.5)	33.0 (4.4)				V3 vs. CON	4.5 (3.1 to 5.90)	<0.001
Hospital anxiety and depression scale								
Anxiety subscale								
CON	6.0 (3.5)	7.0 (3.2)	<0.005	0.448	0.295			
M1	5.1 (2.8)	3.3 (2.0)				M1 vs CON	−3.0 (−4.5 to 1.5)	<0.001
M3	7.9 (4.5)	4.7 (3.5)				M3 vs CON	−3.8 (−5.3 to 2.3)	<0.001
V1	5.3 (4.1)	3.6 (2.9)				V1 vs CON	−2.8 (−4.2 to 1.3)	<0.005
V3	5.3 (1.7)	3.4 (2.1)				V3 vs CON	−3.0 (−4.5 to 1.6)	<0.001
Depression subscale								
CON	6.9 (3.5)	6.4 (3.2)	0.214	0.064	0.500		N/A	N/A
M1	6.3 (2.2)	4.9 (1.8)						
M3	10.9 (1.9)	8.9 (2.3)						
V1	6.3 (3.4)	4.8 (2.3)						
V3	7.5 (4.7)	5.6 (4.2)						
Pittsburgh sleep quality index								
CON	11.0 (3.5)	11.6 (4.2)	0.278	0.742	0.328		N/A	N/A
M1	10.9 (2.1)	9.1 (2.1)						
M3	9.7 (4.3)	7.7 (3.4)						
V1	9.9 (3.5)	6.6 (3.5)						
V3	13.1 (3.5)	9.3 (3.9)						

Data are expressed as mean (standard deviation). Data were analyzed by generalized estimating equations model with baseline measurements as covariates. Closed test procedure with Holm-Bonferroni correction was used in the post hoc analysis. CON, stretching exercise control group, M1, moderate-intensity once-a-week walking group, M3, moderate-intensity thrice-a-week walking group, V1, vigorous-intensity once-a-week walking group, and V3, vigorous-intensity thrice-a-week walking group.

A significant group-by-time interaction effect was observed in the HADS anxiety subscale (*p* < 0.005). After the 12-week walking intervention, the severity of anxiety was significantly reduced in M1, M3, V1, and V3 compared with CON [adjusted mean difference (95% CI): M1 vs. CON: −3.0 (−4.5, −1.5), *p* < 0.001; M3 vs. CON: −3.8 (−5.3, −2.3), *p* < 0.001; V1 vs. CON: −2.8 (−4.2, −1.3), *p* < 0.005; and V3 vs. CON: −3.0 (−4.5, −1.6), *p* < 0.001] (Table 2).

No significant group-by-time interaction effects were observed in the other secondary outcomes including self-report cognitive performance measured by CSRQ, depressive symptoms measured by HADS depression subscale, and subjective sleep quality and quantity measured by PSQI at the post-intervention assessment (Table 2).

### 3.4 Adverse events

No adverse events were reported during the 12-week intervention period.

## 4 Discussion

The present study preliminarily investigated the effects of 12-week walking exercise interventions at different exercise frequencies and intensities on improving cognitive performance of middle-aged and older adults with MCI. Our results demonstrated that the 12-week walking interventions at moderate and vigorous intensities and at once-a-week and thrice-a-week frequencies had similar effects on improving global cognitive performance measured by HK-MoCA in middle-aged and older adults with MCI. Moreover, there were reductions in anxiety severity and improvements in cardiorespiratory fitness. No adverse events were reported during the study period.

The WHO global action plan on physical activity 2018–2030 aims to achieve a relative 15% reduction in the global prevalence of physical inactivity (World Health Organization 2019). The WHO physical activity guidelines were set up in accordance with the WHO Handbook for Guideline Development. The WHO guideline development group first identified several systematic reviews and meta-analyses related to physical activity and cognitive health. There were three meta-analyses that summarized the beneficial effects of physical activity for maintaining and improving general cognitive performance and domain-specific cognitive function in healthy elders (Rathore and Lom 2017; Brasure et al., 2018; Engeroff et al., 2018). A study by Northey and coworkers reported that physical exercise improved cognitive function in older adults over 50, regardless of the cognitive status of participants (Northey et al., 2018). Other studies identified that exercise could confer protective effects on cognitive health in various disease populations, including patients with Parkinson’s disease, schizophrenia, and substance use disorders (Firth et al., 2017; Stuckenschneider et al., 2019; Ashdown-Franks et al., 2020). Overall, these studies demonstrated that physical activity could benefit cognitive performance in different populations. Based on these studies, the WHO recommended that performing regular physical activity could confer beneficial effects including improving cognitive performance (Bull et al., 2020). The WHO physical activity guidelines have been widely adopted by numerous countries and regions worldwide. However, there is a lack of studies that have fully investigated the therapeutic effects of exercise intervention programs adopting the WHO guidelines on improving cognition in the MCI population. The present study found that walking interventions adopting the WHO physical activity guidelines were feasible and effective in improving cognitive performance in middle-aged and older adults with MCI. Our findings have important public health implications for MCI management and support the global use of the WHO physical activity guidelines for enhancing cognitive health.

As part of the overall management, the updated AAN guidelines recommend patients with MCI to perform exercise regularly. However, the guidelines do not include any specific recommendations on exercise intensity, likely due to the lack of rigorous evidence on the effects of different exercise intensities (Petersen et al., 2018). Although a few meta-analyses have reported that moderate-intensity and vigorous-intensity exercise have similar effect sizes on improving global cognitive function and executive function, few studies have directly compared the beneficial effects of different exercise intensities (Northey et al., 2018; Chen et al., 2020). In the present study, we directly compared the effects of moderate-intensity versus vigorous-intensity walking training on improving cognition in MCI patients. Our results showed that both moderate and vigorous walking training significantly improved the cognitive performance of MCI patients. Importantly, the improvements in global cognitive performance in all walking groups were ≥4 as measured in HK-MoCA, which exceed the MCID (Feeney et al., 2016; Kopecek et al., 2017). Notably, MCID is commonly regarded as the minimum patient-reported change representing a significant improvement in a specific disease condition, such as increased cognitive performance in MCI patients. Moreover, such improvements are also associated with general health benefits including better quality of life and mental health (Cook 2008). For instance, Wong and colleagues reported the MCID of MoCA was associated with significantly improved quality of life in patients with aneurysmal subarachnoid hemorrhage history (Wong et al., 2017). In the present study, the within-group improvement score in HK-MoCA was 6 [95% CI: (4.42, 7.58)], 5 (3.42, 6.58), 4.38 (2.9, 5.85), and 5.75 (4.27, 7.23) in M1, M3, V1, and V3, respectively, whereas the score of 0.86 in CON (−0.72, 2.43) was lower than the MCID. Our study demonstrated that 12 weeks of walking exercise adopting the WHO physical activity guidelines can induce a clinically meaningful improvement in the cognitive performance of MCI patients. Future studies should include a long-term follow-up period to examine the translational effects of the exercise-induced MCID of MoCA on general health outcomes such as health-related quality of life and the conversion rate of MCI to dementia.

Besides exercise intensity, another important exercise parameter is exercise frequency. The updated AAN guidelines recommend MCI patients should exercise twice a week for cognitive benefits (Petersen et al., 2018). These updated guidelines were based on two exercise intervention studies by Nagamatsu et al. and Suzuki et al. The study by Nagamatsu et al. evaluated the effectiveness of a 6-month twice-weekly resistance training program for improving cognitive performance and brain plasticity in 86 females with probable MCI. They reported the twice-weekly resistance training improved performance in several cognitive domains including selective attention and associative memory (Nagamatsu et al., 2012). The other study by Suzuki et al. showed a 6-month twice-weekly multicomponent exercise intervention significantly improved logical memory and maintained general cognitive function in 100 older adults with MCI (Suzuki et al., 2013). However, it is important to note that both studies focused on the effectiveness of the exercise intervention without explicitly examining exercise frequency as an independent variable. Therefore, the optimal or minimal exercise frequency required for MCI patients to gain the maximal cognitive benefits remains unknown. Recently, emerging evidence showed that low-frequency exercise (e.g., weekly) might be beneficial for reducing all-cause, cardiovascular, and cancer mortality risks (O’Donovan et al., 2017). Intriguingly, Liu-Ambrose et al. reported that once-a-week resistance training significantly improved executive function in 155 healthy seniors (Liu-Ambrose et al., 2010). Considering the above studies and the lack of evidence, we preliminarily explored the role of exercise frequency (once-a-week versus thrice-a-week) on improving cognition in MCI patients. Our results demonstrated that both once-weekly and thrice-weekly walking training significantly improved the cognitive performance of MCI patients. Our findings suggest that more flexible recommendations on the frequency of physical activity could be given to MCI patients, as all intervention groups showed similar benefits. Time commitment is considered to be one of the main barriers preventing people from engaging in regular exercise (Hoare et al., 2017). Middle-aged and older adults with MCI, especially those who have limited time during the week, could still benefit from once-a-week aerobic-type physical activity at the appropriate intensity according to the recommendations by the WHO, namely 75-min vigorous-intensity or 150-min moderate-intensity aerobic-type physical activity.

This study had some limitations that should be noted. The 12-week interventions were conducted in a laboratory-based environment, which might mean the study findings cannot be fully generalizable to outdoor exercise training. Nevertheless, our experimental protocol was conducted under a rigorously controlled environment with participants in all groups undergoing the interventions in the same environmental conditions. Our results clearly showed that walking exercise performed at different intensities and frequencies can improve cognitive performance after controlling for several confounding factors that are known to affect cognition, such as noise and atmospheric pollutants (Clark and Paunovic 2018; Cassilhas et al., 2021). Another potential issue is that our participants received individual training rather than group-based training. Indeed, loneliness has been demonstrated to be a strong independent risk factor of cognitive decline and dementia, whereas social interaction can exert protective effects (Holwerda et al., 2014; Lee et al., 2017). As our aim was to dissect the roles of exercise intensity and frequency on cognition, we chose individual over group-based training because some participants might respond differently in a group setting or some participants might become marginalized, which would introduce extra confounding effects. Future studies are needed to compare the effects of group versus individual training on cognition in older adults with MCI. Small sample size, which is common in pilot studies, limited the robustness of the findings of the present study. Further studies with large sample size and adequate statistical power are warranted to validate our preliminary results.

## 5 Conclusion

In conclusion, moderate or vigorous intensity walking exercise performed once- or thrice-weekly showed similar effects on improving cognitive performance in middle-aged and older adults with MCI. The 12-week walking exercise interventions also reduced anxiety severity and improved cardiorespiratory fitness.

## Data Availability

The raw data supporting the conclusion of this article will be made available by the authors, without undue reservation.
